# Extracellular polymeric substance (EPS)-degrading enzymes reduce staphylococcal surface attachment and biocide resistance on pig skin *in vivo*

**DOI:** 10.1371/journal.pone.0205526

**Published:** 2018-10-10

**Authors:** Jeffrey B. Kaplan, Kevin D. Mlynek, Hashani Hettiarachchi, Yonas A. Alamneh, Lionel Biggemann, Daniel V. Zurawski, Chad C. Black, Charles E. Bane, Robert K. Kim, Mark S. Granick

**Affiliations:** 1 Department of Biology, American University, Washington, District of Columbia, United States of America; 2 Wound Infections Department, Walter Reed Army Institute of Research, Silver Spring, Maryland, United States of America; 3 Department of Surgery, Rutgers New Jersey Medical School, Newark, New Jersey, United States of America; University Medical Center Utrecht, NETHERLANDS

## Abstract

Staphylococcal extracellular polymeric substances (EPS) such as extracellular DNA (eDNA) and poly-*N-*acetylglucosamine surface polysaccharide (PNAG) mediate numerous virulence traits including host colonization and antimicrobial resistance. Previous studies showed that EPS-degrading enzymes increase staphylococcal biocide susceptibility *in vitro* and *in vivo*, and decrease virulence in animal models. In the present study we tested the effect of EPS-degrading enzymes on staphylococcal skin colonization and povidone iodine susceptibility using a novel *in vivo* pig model that enabled us to colonize and treat 96 isolated areas of skin on a single animal *in vivo*. To quantitate skin colonization, punch biopsies of colonized areas were homogenized, diluted, and plated on agar for colony forming unit enumeration. Skin was colonized with either *Staphylococcus epidermidis* or *Staphylococcus aureus*. Two EPS-degrading enzymes, DNase I and the PNAG-degrading enzyme dispersin B, were employed. Enzymes were tested for their ability to inhibit skin colonization and detach preattached bacteria. The effect of enzymes on the susceptibility of preattached *S*. *aureus* to killing by povidone iodine was also measured. We found that dispersin B significantly inhibited skin colonization by *S*. *epidermidis* and detached preattached *S*. *epidermidis* cells from skin. A cocktail of dispersin B and DNase I detached preattached *S*. *aureus* cells from skin and increased their susceptibility to killing by povidone iodine. These findings suggest that staphylococcal EPS components such as eDNA and PNAG contribute to skin colonization and biocide resistance *in vivo*. EPS-degrading enzymes may be a useful adjunct to conventional skin antisepsis procedures in order to further reduce skin bioburden.

## Introduction

*Staphylococcus aureus* and *Staphylococcus epidermidis* are among the most common bacteria isolated from infections of surgical wounds and surgically-implanted medical devices [1, 2]. Both species secrete extracellular polymeric substances (EPS) including surface- and cell wall-associated proteins, capsular polysaccharides, and double-stranded DNA. EPS contributes to staphylococcal virulence by forming a matrix that surrounds the cells, protecting them from killing by antimicrobial agents and host defenses, and helping them attach to host tissues and abiotic surfaces [3]. Two major components of the staphylococcal EPS matrix are poly-*N*-acetylglucosamine surface polysaccharide (PNAG) [4] and extracellular DNA (eDNA) [5]. In *S*. *epidermidis*, PNAG has been shown to mediate resistance to killing by human polymorphonuclear leukocytes, antimicrobial peptides, and the cationic surfactant cetylpyridinium chloride (CPC) *in vitro* [6, 7], and attachment of bacteria to human umbilical vein endothelial cells and human urinary bladder epithelial cells *in vitro* [8]. In *S*. *aureus*, PNAG has been shown to mediate resistance to killing by 5% ethanol in *in vitro* [9], and adhesion of bacteria to human nasal epithelial cells in a mouse model [10]. eDNA has been shown to mediate *S*. *aureus* resistance to killing by CPC *in vitro* [6]. Both PNAG and eDNA also contribute to intercellular adhesion and biofilm formation by both *S*. *epidermidis* and *S*. *aureus in vitro* [3, 6].

Enzymes that degrade PNAG and eDNA (dispersin B and DNase I, respectively) have been shown to reduce staphylococcal intercellular adhesion, surface attachment, biofilm formation and antimicrobial resistance *in vitro* [6, 11, 12]. Neither enzyme alone affects the growth or viability of *S*. *aureus in vitro* [12, 13]. *In vivo*, DNase I was shown to significantly increase the survival of *S*. *aureus*-infected *Caenorhabditis elegans* nematodes treated with tobramycin compared with nematodes treated with tobramycin alone [12], and dispersin B was shown to eradicate *S*. *aureus* port-related bloodstream infections in catheterized sheep when used in combination with teicoplanin as a catheter lock solution [14], and to resist *S*. *aureus* colonization as efficiently as chlorhexidine-silver sulfadiazine-coated catheters in an *in vivo* rabbit subcutaneous implant model [15]. No studies that measured the activity of dispersin B and DNase I on staphylococcal skin colonization *in vivo*, or against *S*. *epidermidis in vivo*, have been reported.

In the present study we measured the effect of dispersin B and DNase I on *S*. *aureus* and *S*. *epidermidis* skin colonization and susceptibility to killing by povidone iodine in an *in vivo* pig model. Pigs are considered to be suitable models for human cutaneous colonization because porcine skin is anatomically and physiologically similar to human skin [16, 17]. Here we show that EPS-degrading enzymes inhibit staphylococcal skin colonization, detach preattached bacteria from skin, and sensitize preattached bacteria to povidone iodine killing *in vivo*.

## Materials and methods

### Bacteria

The bacteria used in this study were *Staphylococcus epidermidis* strain NJ9712 [11], *S*. *epidermidis* strain 5 [18], and *Staphylococcus aureus* strain MZ100 [19]. *S*. *epidermidis* strains NJ9712 and 5 were isolated from hospitalized patients with implant-related infections. *S*. *aureus* strain MZ100 is a laboratory strain derived from strain 8325–4 [20]. All strains were stored at -80°C in 20% dimethyl sulfoxide and cultured at 37°C on sheep blood agar.

### Enzymes and biocides

Recombinant dispersin B (also known as DspB), a 42-kDa PNAG-specific glycosidase [21], was obtained from Kane Biotech (Winnipeg, Manitoba, Canada). Recombinant human DNase I, a 37-kDa nuclease with preference for double-stranded DNA, was obtained from Genentech, Inc. (South San Francisco, California). Povidone-iodine 10% solution (Betadine) was purchased from Purdue Pharma (Stamford, Connecticut).

### Preparation of bacterial inocula

A loopful of cells from a 24-h-old blood agar plate was transferred to a tube containing 200 μl of Tryptic Soy broth supplemented with 6 g l^-1^ yeast extract and 8 g l^-1^ glucose (TSBYG). The cells were dispersed by vortex agitation, diluted 1:100 in fresh TSBYG, and then passed through a 5-μm pore-size syringe filter to remove large clumps of cells [22]. The resulting inocula contained 5–10 × 10^7^ colony forming units (c.f.u.) ml^-1^ as determined by dilution plating. In some experiments inocula were supplemented with EPS-degrading enzymes as described below.

### Pig skin colonization model

#### Ethical statement

Research was conducted in an AAALACi accredited facility in compliance with the Animal Welfare Act and other federal statutes and regulations relating to animals and experiments involving animals, and adheres to principles stated in the National Research Council publication *Guide for the Care and Use of Laboratory Animals* (The National Academies Press, Washington, District of Columbia, 2011 ed.). The animal protocol was approved by the Walter Reed Army Institute of Research Institutional Animal Care and Use Committee (Protocol # 13-BRD-16LS).

#### Animals

Female Yorkshire pigs (20–25 kg) were used in this study. Animals were housed individually and allowed to acclimate for one week prior to initiating the experimental procedure. One animal was used in each experiment. Animals were anesthetized with an IM injection of ketamine/xylazine/atropine and maintained under anesthesia throughout the procedure (3–4 h) by means of mask inhalation of isoflurane/oxygen. After the procedure, animals were euthanized with a pentobarbital-containing euthanasia solution. A total of 16 animals was used in this study.

#### Preparation of pig skin

**Fig 1:**
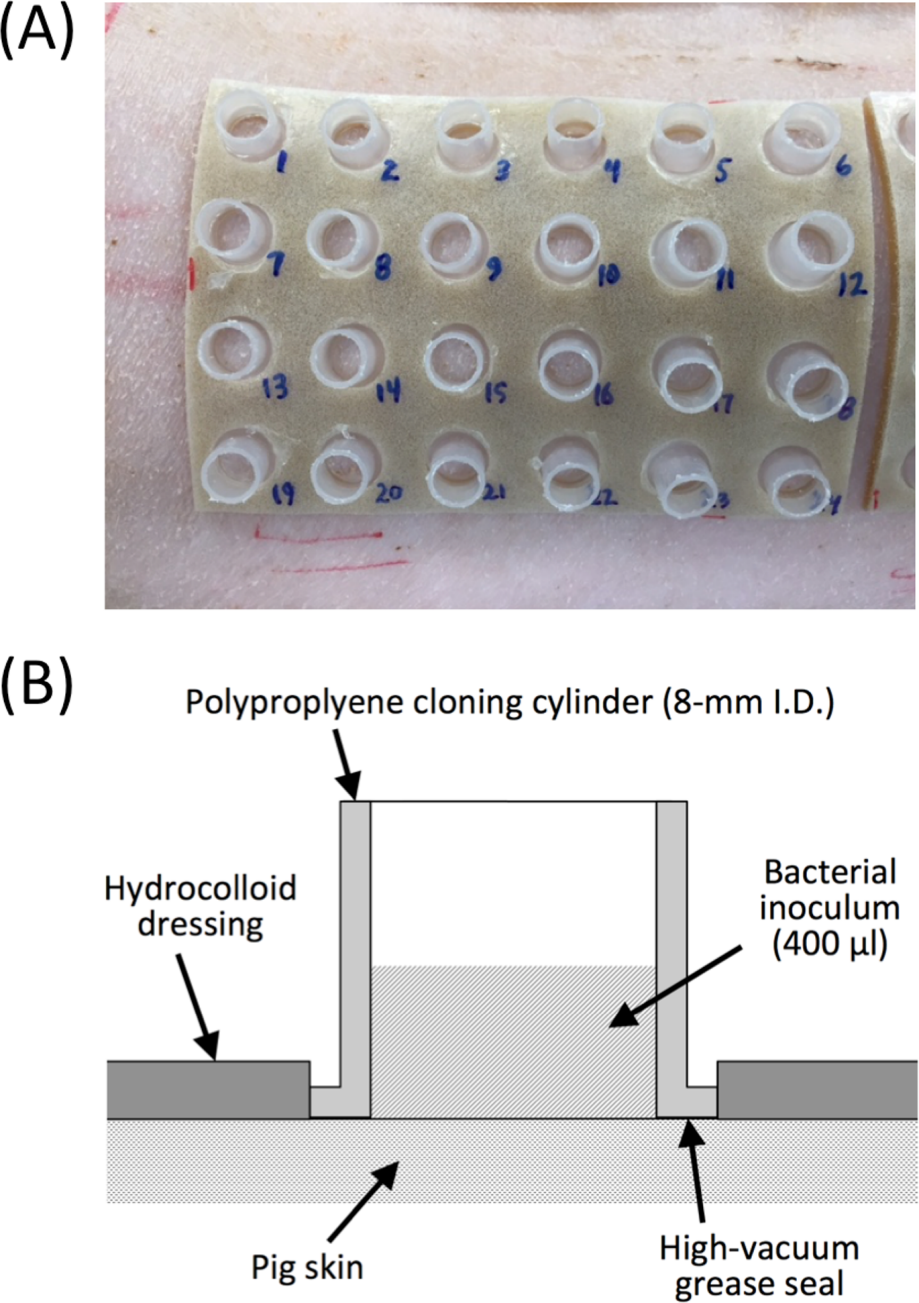
System for measuring attachment of bacteria to porcine skin. (a) Photograph of 24 skin inoculation sites arrayed on a 90 × 140-mm hydrocolloid dressing. Each inoculation site was isolated by means of a polypropylene cloning cylinder attached to the skin with high vacuum grease. A total of four dressings were placed on each animal, two on each side. (b) Schematic of a single skin inoculation site in cross section.

The back and flanks of the pig were shaved with an animal clipper, fine shaved with an electric razor, and cleaned thoroughly with a soft 2” paint brush. Four sheets of DuoDERM CGF hydrocolloid dressing (ConvaTec, Greensboro, North Carolina) were applied to the skin, two on each side of the animal (Fig 1A). Each dressing was 90 mm × 140 mm in size, and contained 24 12-mm-diam holes arranged in a 4 × 6 array. The holes were precut in the dressing with a 12-mm-diam biopsy punch. A sterile cloning cylinder was placed in each hole and attached to the skin with a thin layer of high vacuum grease (Fig 1B). Cloning cylinders were made by cutting the lid and bottom off of a standard 1.5-ml polypropylene centrifuge tube (catalog number 1615–5500; USA Scientific, Ocala, Florida).

#### Inoculation of pig skin and c.f.u. enumeration

Each cloning cylinder was filled with 400 μl of bacterial inoculum. After 1 or 2 h, the inoculum was aspirated, the cloning cylinder was removed, and a 6-mm punch biopsy was taken from the center of the inoculation site. The biopsy sample was transferred to a 15-ml conical centrifuge tube containing 5 ml of phosphate buffered saline (PBS) and mixed by high-speed vortex agitation for 5 sec to remove loosely adherent cells. The biopsy sample was then transferred to 4-ml polyethylene grinding vial equipped with a 9.5-mm stainless steel grinding ball (catalog number 97007–694; VWR International, Radnor, Pennsylvania). The grinding vial was filled with 400 μl of PBS and stored on ice until all the biopsy samples were collected and processed.

To quantitate skin colonization, biopsies were homogenized at full speed for 2 min in a high-throughput reciprocating motion homogenizer (catalog number 12621–164; VWR International). Dilutions of the homogenate (10^−1^, 10^−2^, 10^−3^ and 10^−4^) were plated on blood agar and incubated at 37°C for 16 h. Colony forming unit (c.f.u.) mm^-2^ values were calculated based on the colony counts. The lower limit of detection in this assay was 15 c.f.u. mm^-2^.

#### Enzyme and biocide treatments

To measure the effect of EPS-degrading enzymes on skin colonization, inocula were supplemented with 10 μg ml^-1^ dispersin B, 10 μg ml^-1^ DNase I, or 10 μg ml^-1^ of both enzymes. Control inocula were left unsupplemented. After 1 h, skin colonization was quantitated as described above.

To measure the ability of EPS-degrading enzymes to detach preattached bacteria from the skin, bacteria were allowed to colonize the skin for 1 h and the inoculum was aspirated. Cloning cylinders were then filled with 400 μl of enzyme buffer (10 mM HEPES, pH 7.0, 1 mM MgCl_2_, 1 mM CaCl_2_) or enzyme buffer containing 10 μg ml^-1^ dispersin B, 10 μg ml^-1^ DNase I, or 10 μg ml^-1^ of both enzymes. After 20 min, enzymes were aspirated and skin colonization was quantitated as described above.

To measure the effect of EPS-degrading enzymes on the susceptibility of preattached bacteria to killing by povidone iodine, bacteria were allowed to colonize the skin for 1 h and the inoculum was aspirated. Cloning cylinders were then filled with 400 μl of enzyme buffer, or enzyme buffer containing a mixture of 10 μg ml^-1^ dispersin B plus 10 μg ml^-1^ DNase I. After 10 min, buffer or enzyme were aspirated and 400 μl of 0.4% povidone iodine solution was transferred to the cloning cylinder. Control cylinders received 400 μl of water. After 5 min, the povidone iodine solution or water were aspirated, and skin colonization was quantitated as described above.

### Statistical analyses

Mean c.f.u. mm^-2^ values and standard deviations were calculated from sextuplet cloning cylinders from a single animal in each experiment. Figures show a single representative experiment for each assay. All of the observed statistically significant differences in mean c.f.u. mm-2 values shown in the figures were observed in at least one additional experiment. The significance of differences between mean c.f.u. mm^-2^ values was measured using a 2-tailed Student’s *t*-test for pairwise comparisons, and one-way ANOVA with Tukey’s post hoc analysis for comparison of more than two groups. A *P* value of <0.05 was considered significant.

## Results

### Experimental skin colonization model

An *in vivo* porcine skin model was developed to assess the bioburden removal efficacy of EPS-degrading enzymes on living skin (Fig 1). In this model, 96 areas of skin (8-mm diam each) were isolated by means of polypropylene cloning cylinders attached to the skin with high vacuum grease and held in place by a hydrocolloid dressing. The cloning cylinders could easily be filled with inocula, aspirated and treated with enzymes and biocides with no well-to-well cross contamination. The level of background colonization on uninoculated skin was measured by sampling 12 areas of skin from two different animals. Background skin colonization levels were <200 c.f.u. mm^-2^, with the majority of sampled sites exhibiting no detectable background colonization (<15 c.f.u. mm^-2^). Background colonization levels were sufficiently low so that no additional skin preparation, besides shaving and brushing, was performed prior to skin inoculation. As a result, the host immune functions and resident microflora remained intact during the colonization process.

**Fig 2:**
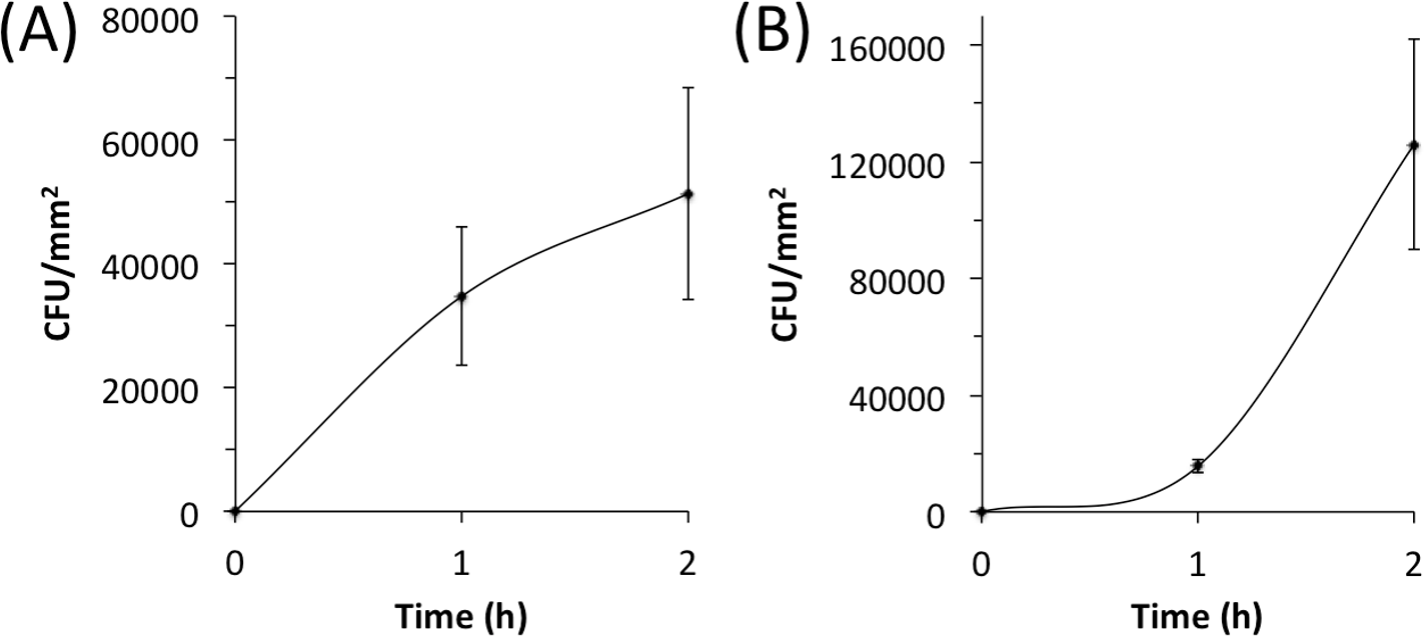
**Attachment of *S*. *epidermidis* strain 5 (a) and strain NJ9712 (b) to porcine skin.** A volume of 400 μl of bacteria (5–10 × 10^7^ c.f.u. ml^-1^) was inoculated onto the skin. After 1 or 2 h, inoculation sites were aspirated, excised with a biopsy punch, rinsed with PBS, homogenized, diluted, and plated for c.f.u. enumeration. Values indicate mean c.f.u. mm^-2^ counts for six inoculation sites from a single animal and error bars indicate sd.

To validate the pig model we colonized skin with *S*. *epidermidis* strains 5 and NJ9712, and quantitated colonization after 1 and 2 h (Fig 2). Both strains colonized skin in a time-dependent manner, achieving 2–4 × 10^4^ c.f.u. mm^-2^ after 1 h and 0.5–1 × 10^5^ c.f.u. mm^-2^ after 2 h. The kinetics of attachment suggest that *S*. *epidermidis* cells are irreversibly attaching to the skin surface and replicating during the 2-h colonization period [23, 24].

### Effect of enzymes on *S*. *epidermidis* skin colonization

**Fig 3:**
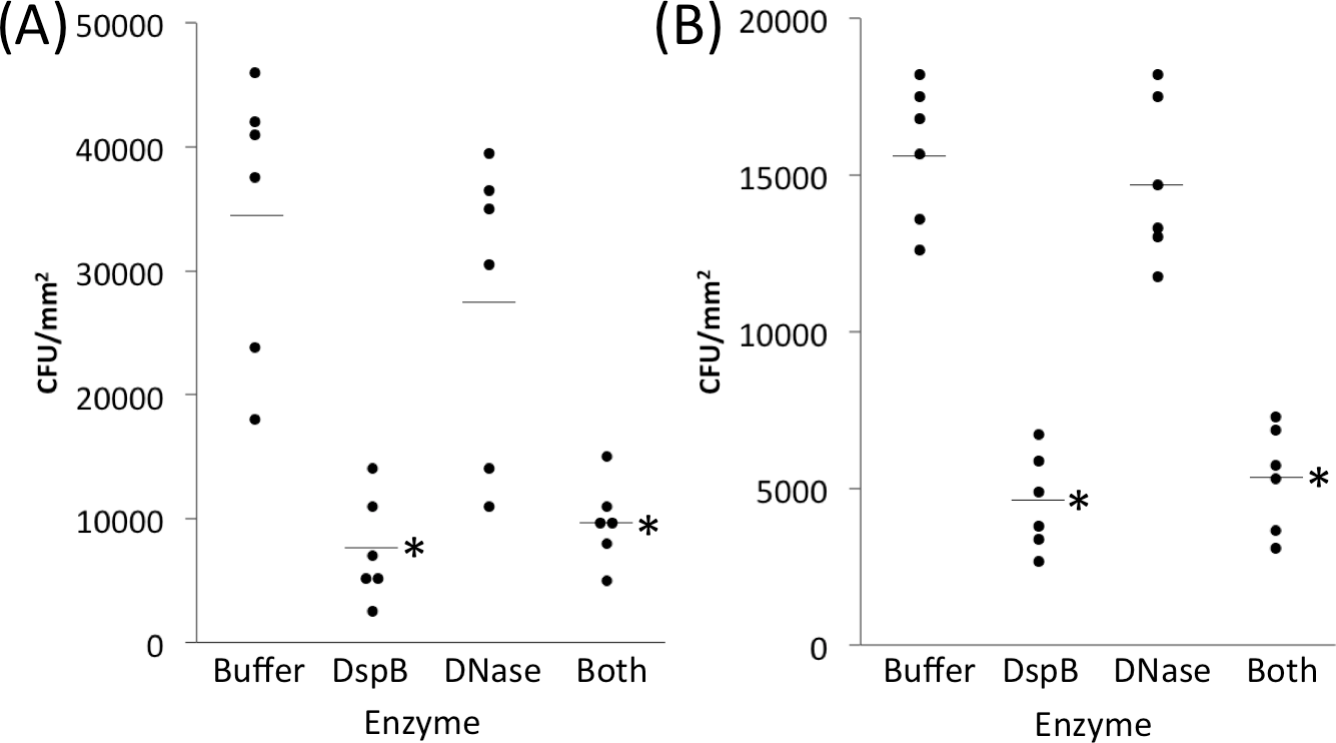
**Dispersin B (DspB) inhibits attachment of *S*. *epidermidis* strains 5 (a) and NJ9712 (b) to porcine skin.** Attachment assays were carried out for 1 h as described in Fig 2 except that some inocula were supplemented with 10 μg ml^-1^ dispersin B (DspB), 10 μg ml^-1^ DNase I (DNase), or 10 μg ml^-1^ of both enzymes (Both). Enzyme buffer (Buffer) served as a control. Dots show individual c.f.u. mm^-2^ values from six inoculation sites from a single animal for each condition. Horizontal lines indicate means. *, significantly different from buffer control (*P* < 0.005) as determined by one-way ANOVA with Tukey’s post hoc analysis.

To determine whether EPS-degrading enzymes could inhibit attachment of *S*. *epidermidis* cells to pig skin, we allowed *S*. *epidermidis* strains 5 and NJ9712 to colonize skin for 1 h in the presence of 10 μg ml^-1^ dispersin B, 10 μg ml^-1^ DNase I, or 10 μg ml^-1^ of both enzymes (Fig 3). Compared to the no-enzyme control, dispersin B, or a mixture of both enzymes, reduced c.f.u. mm^-2^ values from >3 × 10^4^ to <1 × 10^4^ for strain 5, and from >1.5 × 10^4^ to 5 × 10^3^ for strain NJ9712. The amount of inhibition was 66–78% compared to the enzyme buffer control and was highly significant for both strains for both dispersin B and the enzyme mixture (*P* <0.005) as determined by one-way ANOVA with Tukey’s post hoc analysis. DNase I itself had no significant effect on skin colonization by either strain, exhibiting 6–20% colonization inhibition.

**Fig 4:**
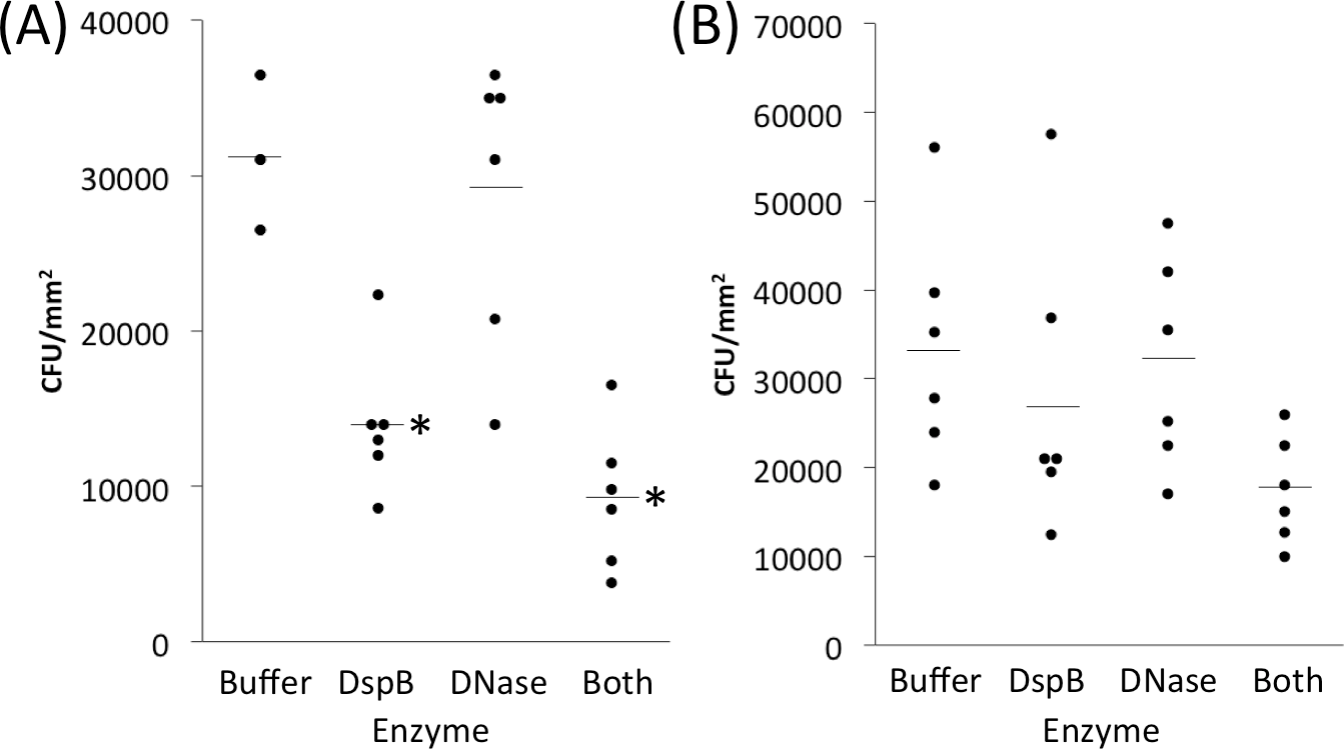
EPS-degrading enzymes detach *S*. *epidermidis* from porcine skin. *S*. *epidermidis* strains 5 (a) and NJ9712 (b) were allowed to attach for 1 h. Inoculation sites were then aspirated and treated with 10 μg ml^-1^ dispersin B (DspB), 10 μg ml^-1^ DNase I (DNase), or 10 μg ml^-1^ of both enzymes (Both) for 20 min. Enzyme buffer (Buffer) served as a control. Dots show individual c.f.u. mm^-2^ values from three to six inoculation sites from a single animal for each condition. In the experiment shown in panel A, three Buffer samples were lost during processing. Horizontal lines indicate means. *, significantly different from buffer control (*P* < 0.005) as determined by one-way ANOVA with Tukey’s post hoc analysis.

To test whether EPS-degrading enzymes could detach preattached *S*. *epidermidis* cells from pig skin, we allowed *S*. *epidermidis* strains 5 and NJ9712 to colonize for 1 h. We then removed the inoculum and treated the colonized areas for 20 min with 10 μg ml^-1^ dispersin B, 10 μg ml^-1^ DNase I, or a mixture of 10 μg ml^-1^ of both enzymes (Fig 4). Control areas were treated with enzyme buffer alone. Compared to the buffer control, dispersin B, or a mixture of both enzymes, significantly reduced c.f.u. mm^-2^ values on the skin by 55–71% (from >3 × 10^4^ to 1 × 10^4^) for strain 5 (Fig 4A; *P* <0.005; one-way ANOVA with Tukey’s post hoc analysis). A mixture of both enzymes reduced c.f.u. mm^-2^ values by 48% from >3 × 10^4^ to <2 × 10^4^ for strain NJ9712 (Fig 4B), but this difference was not statistically significant. DNase I itself exhibited no significant detaching activity against either strain (5–8% detachment efficiency).

### Effect of enzymes on *S*. *aureus* skin colonization and biocide resistance

**Fig 5:**
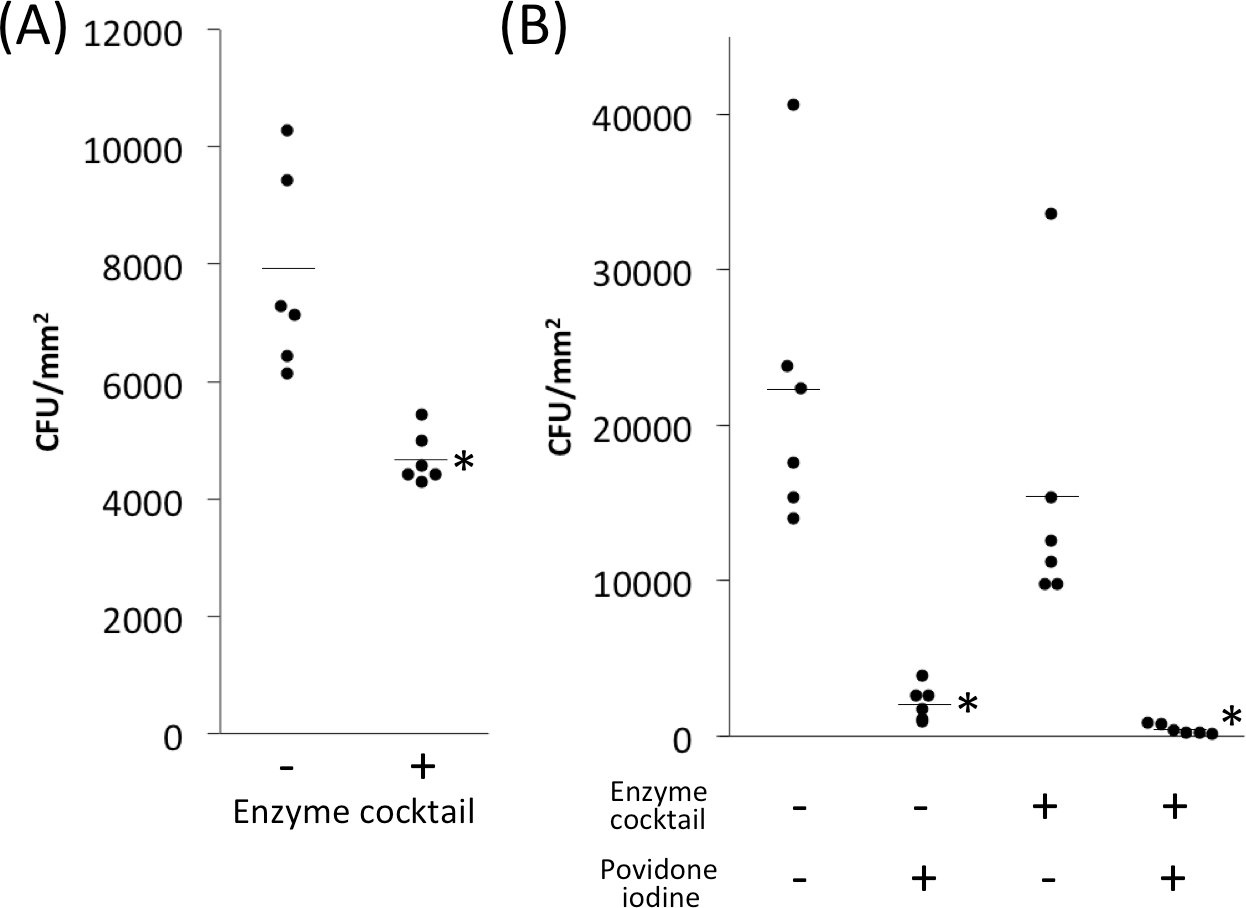
EPS-degrading enzyme cocktail detaches *S*. *aureus* strain MZ100 from porcine skin and sensitizes it to killing by povidone iodine. (a) Bacteria were allowed to attach to skin for 1 h. Inocula were then aspirated and skin was treated with enzyme cocktail (10 μg ml^-1^ dispersin B plus10 μg ml^-1^ DNase I) for 20 min. Control skin was treated with enzyme buffer alone. Dots show individual c.f.u. mm^-2^ values from six inoculation sites from a single animal for each condition, and horizontal lines indicate means. *, *P* = 0.002 compared to no enzyme control by two-tailed *t*-test. (b) *S*. *aureus* strain MZ100 was allowed to attach to porcine skin for 1 h. Inoculation sites were then aspirated and treated with enzyme cocktail (or enzyme buffer control) for 10 min, followed by 0.4% povidone iodine (or water control) for 5 min. Dots show individual c.f.u. mm^-2^ values from six inoculation sites from a single animal for each condition. *, significantly different from enzyme buffer/no drug control (*P* < 0.005) as determined by one-way ANOVA with Tukey’s post hoc analysis.

To test whether EPS-degrading enzymes could detach preattached *S*. *aureus* cells from skin, we allowed *S*. *aureus* strain MZ100 to colonize skin for 1 h, aspirated the inoculum, and then treated the preattached cells for 20 min with an enzyme cocktail containing 10 μg ml^-1^ each of dispersin B and DNase I (Fig 5A). Control skin was treated with enzyme buffer alone. The enzyme-treated skin contained 40% fewer cells than the buffer-treated skin (4.7 × 10^3^ c.f.u. mm^-2^ for enzyme treatment versus 7.8 × 10^3^ c.f.u. mm^-2^ for buffer control. This difference was significant (*P* = 0.002) by two-tailed *t*-test.

To test whether EPS-degrading enzymes can sensitize preattached *S*. *aureus* cells to killing by povidone iodine, we allowed *S*. *aureus* strain MZ100 to colonize skin for 1 h, aspirated the inoculum, and then treated the preattached cells for 10 min with an enzyme cocktail containing 10 μg ml^-1^ each of dispersin B and DNase I, or enzyme buffer alone as a control, followed by a 5-min treatment with 0.4% povidone iodine solution, or water as a control (Fig 5B). Skin treated with enzymes alone contained 31% fewer cells than buffer control (1.5 × 10^4^ c.f.u. mm^-2^ for enzyme treatment versus 2.2 × 10^4^ c.f.u. mm^-2^ for buffer control; not significant), whereas skin treated with povidone iodine alone contained 90% fewer cells than water control (2.2 × 10^3^ c.f.u. mm^-2^ for povidone iodine treatment versus 2.2 × 10^4^ c.f.u. mm^-2^ for water control; *P* < 0.005 by one-way ANOVA with Tukey’s post hoc analysis). Skin treated with a combination of enzymes and povidone iodine contained 80% fewer cells than skin treated with povidone iodine alone (4.3 × 10^2^ c.f.u. mm^-2^ for combination treatment versus 2.2 × 10^3^ c.f.u. mm^-2^ for povidone iodine alone) but this difference was not statistically significant. Under the conditions tested, the enzyme cocktail increased the killing activity of povidone iodine from 90% to 98% (0.7 log units).

## Discussion

Skin microflora play an active role in host immune defense by inhibiting pathogen growth [25] and enhancing host innate and adaptive immunity [26, 27]. However, skin microbes may become opportunistic pathogens when the cutaneous barrier is broken during skin-penetrating medical procedures such as surgery [28, 29]. It has been shown that skin microbes can persist in dermal tissue even after standard presurgical skin preparation [30] and can be subsequently transferred to the underlying tissue through a physical breach of the epidermis [29]. Surgical site infection is one of the most common healthcare-associated infections [31] and a major cause of morbidity and mortality among surgical patients [32].

*Staphylococcus aureus* and *S*. *epidermidis* are among the most common pathogens isolated from surgical site infections. Both species secrete extracellular polymeric substances (EPS) such as eDNA and PNAG polysaccharide which mediate numerous virulence traits including host colonization and antimicrobial resistance [3, 4]. Previous studies showed that DNase I and the PNAG-degrading enzyme dispersin B increase staphylococcal biocide susceptibility and decrease virulence in animal models [12, 14, 15]. Since no studies have measured the activity of DNase I and dispersin B on staphylococcal skin colonization *in vivo*, or against *S*. *epidermidis in vivo*, in the present study we tested the effect of these enzymes on *S*. *aureus* and *S*. *epidermidis* skin colonization and biocide susceptibility in an *in vivo* pig model.

To assess the activity of DNase I and dispersin B on staphylococcal skin colonization, we developed a novel porcine model that enabled us to colonize and treat 96 individual areas of skin on a single, live pig (Fig 1). Pigs are commonly used as a model for human cutaneous colonization and infection because pig skin is very similar to human skin in terms of anatomy, thickness, hair follicle content, pigmentation, lipid composition, immunological components and bacterial microflora [33–35]. In our model, skin was not treated with detergents or antiseptics prior to inoculation in order to maintain the natural skin immune functions and resident microflora. In addition, skin was inoculated with bacteria that had been passed through a 5-μm pore-size filter to increase the uniformity of the inoculum, and bacteria were allowed to colonize skin in a biofilm-promoting rich medium. We expected that these steps would increase the accuracy and robustness of the model. Pilot time-course skin colonization assays using two clinical strains of *S*. *epidermidis* (strains 5 and NJ9712) showed that the pig model yielded c.f.u. measurements with enough precision to detect colonization differences with significance levels as low as 5 × 10^−4^ (Fig 2). Interestingly, *S*. *epidermidis* strain 5 colonized skin more rapidly than strain NJ9712, possibly due to the fact that strain 5 produces copious amounts of PNAG [36, 37].

We first tested the ability of dispersin B and DNase I to inhibit skin colonization by *S*. *epidermidis* (Fig 3) and to detach preattached *S*. *epidermidis* cells from skin (Fig 4). We found that dispersin B, or a cocktail of dispersin B and DNase I, significantly inhibited skin colonization by both strains 5 and NJ9712, and a cocktail of dispersin B and DNase I significantly detached preattached cells of strain 5. The amount of inhibition was 66–78% compared to the no enzyme control (Fig 3), and the amount of detachment was 48–71% compared to the no enzyme control (Fig 4). These results are consistent with those of previous *in vitro* studies demonstrating that dispersin B efficiently inhibits and detaches biofilms of *S*. *epidermidis* clinical strains that produce PNAG-dependent biofilms, but not of clinical strains that produce biofilms dependent on the production of cell surface adhesins such as cell wall-anchored proteins and cell wall teichoic acid [38–40]. In addition, PNAG has been shown to mediate attachment of *S*. *epidermidis* to human umbilical vein endothelial cells and human urinary bladder epithelial cells *in vitro* [8]. Also consistent with *in vitro* studies, DNase I had less of an effect on *S*. *epidermidis* skin colonization than dispersin B. However, eDNA production has been shown to be common among *S*. *epidermidis* strains isolated from postsurgical and biomaterial-related orthopedic infections [41], and eDNA production is a common phenotype exhibited by *S*. *epidermidis* strains isolated from ocular infections [42]. Thus, eDNA may play a role in some *S*. *epidermidis* infections but not in skin colonization. Nevertheless, our results clearly demonstrate that PNAG polysaccharide can contribute to skin colonization by *S*. *epidermidis* strains 5 and NJ9712 *in vivo* under the conditions tested.

We also investigated the ability of a cocktail of DNase I and dispersin B to detach *S*. *aureus* cells from skin and render them sensitive to killing by povidone iodine (Fig 5). The strain used was *S*. *aureus* MZ100, a derivative of the well-characterized laboratory strain 8325–4 [20]. We found that the enzyme cocktail reduced *S*. *aureus* skin colonization by 40% (Fig 5A) and increased the killing activity of povidone iodine against preattached cells from 90% to 98% (0.7 log units; Fig 5B). Previous *in vitro* studies carried out in polystyrene microtiter plates showed that DNase I increased the killing activity of povidone iodine, chlorhexidine gluconate and benzalkonium chloride by 4–5 log units against biofilms of *S*. *aureus* strain SH1000 compared to biofilms pretreated with DNase I buffer alone [6, 12]. Therefore, DNase I appears to exhibit less potent biocide potentiating activity against *S*. *aureus* biofilms *in vivo* than *in vitro*. There are several possible explanation for this difference. First, *in vitro* studies were carried out using mature 16-h-old biofilms cultured in polystyrene microtiter plates, whereas the cells analyzed in the present study were allowed to colonize pig skin for only 1 h. Different adhesins may be expressed during the initial stages of skin colonization than in mature biofilms. Also, *S*. *aureus* may form eDNA-dependent biofilms on polystyrene *in vitro*, whereas colonization of pig skin may depend on protein-based adhesins *in vivo* [3]. It is also possible that DNase I and dispersin B exhibit an antagonistic interaction. For example, one recent study showed that a combination of DNase I and dispersin B was significantly less effective in enhancing the antimicrobial efficacy of tobramycin against *S*. *aureus* than the individual enzymes alone [43]. However, a combination of DNase I and dispersin B did exhibit additive biofilm inhibiting activity against *S*. *epidermidis* [12]. More experiments testing individual enzymes against early attaching cells and mature biofilms are needed in order to determine the relative contribution of eDNA and PNAG during different stages of *S*. *aureus* skin colonization.

In summary, our results demonstrate that DNase I and dispersin B modulate staphylococcal skin colonization and biocide resistance *in vivo*, which suggests that eDNA and PNAG contribute to these processes. These findings are consistent with those of previous studies demonstrating that eDNA and PNAG contribute to staphylococcal host colonization and pathogenesis *in vivo* [10, 12, 14, 15, 44] and suggest that EPS-degrading enzymes may be useful adjuncts to conventional presurgical skin antisepsis in order to reduce skin bioburden. Since both eDNA and PNAG are produced by diverse prokaryotic and eukaryotic pathogens [9, 45, 46], DNase I and dispersin B may be effective at mitigating skin colonization of other microorganisms *in vivo* as well.

### Disclaimer

This article has been reviewed by the Walter Reed Army Institute of Research. There is no objection to its presentation and/or publication. The opinions or assertions contained herein are the private views of the authors, and are not to be construed as official, or as reflecting true views of the Department of the Army or the Department of Defense.
